# A choice experiment for testing the energy-efficiency mortgage as a tool for promoting sustainable finance

**DOI:** 10.1007/s12053-022-10035-y

**Published:** 2022-05-04

**Authors:** Federico Dell’Anna, Carlos Marmolejo-Duarte, Marina Bravi, Marta Bottero

**Affiliations:** 1 Department of Regional and Urban Studies and Planning, Politecnico Di Torino, 10125 Turin, Italy; 2 Escola Tècnica Superior d’Arquitectura de Barcelona, Centre de Política de Sòl I Valoracions, Universitat Politècnica de Catalunya, 08028 Barcelona, Spain

**Keywords:** Sustainable energy financing, Energy decision, Stated preference survey, User perception, Housing market

## Abstract

The challenges currently facing the EU in the energy sector include increasing import dependence, limited diversification, high and volatile energy prices, decarbonization, and slow progress in energy efficiency. EU energy policy has provided a wide range of measures to achieve an integrated energy market and sustainability of the building sector. Various incentives and financial instruments have been promoted and financed by governments to help consumers in energy retrofit processes. These include direct investments and fiscal, financial, and market instruments. Public measures have been widely studied but private initiatives have not. In this study, the energy-efficiency mortgage (EEM) is investigated as a credit scheme to attract young people to a real estate market for sustainable buildings, characterized by high sale prices. A choice experiment (CE) was modelled to investigate the preferences of potential young buyers of a new home. The results of this exploratory survey showed an appreciation of the purchase of new A-rated properties according to the Energy Performance Certificate, compared to those that need to be retrofitted or not retrofitted. Consumers like the option of a home energy efficiency renovation being fully managed by a third party (i.e., following the one-stop shop model). The key appeal of energy efficiency for consumers ranges from its ability to support better energy management, to better property value management. The EEM seems to be a promising tool to stimulate investments in energy efficiency and to promote the accessibility and affordability of housing in the sustainable housing market. The results of this study can help private financial institutions to propose appropriate credit plans, which limit the risk of default by the borrower. They can also help public incentive policymakers to propose complementary instruments to EEM.

**Supplementary Information:**

The online version contains supplementary material available at 10.1007/s12053-022-10035-y.

## Introduction

Consumer awareness of issues related to environmental and social sustainability is set to become increasingly important in development strategies determined at governmental level (Assumma et al., 2021; Caprioli et al., 2019). In 2019, Europe’s climate agreement, better known as the European Green Deal, was one of the most important initiatives at global level in the fight against global warming to achieve carbon neutrality (Rivas et al., 2021). The European Commission presented a very detailed action programme, with funding sources that had already been identified for each action and project, for a total budget of more than one trillion euros in investments to be made in the next 10 years. Furthermore, the recent war in Eastern Europe is accelerating the transition towards an energy-independent European Union. Public investment alone would be insufficient to achieve these goals. Therefore, the private sector is a strong point for activating the necessary wave of energy retrofits (Gagliano et al., 2017; Ruggeri et al., 2020). In this context, an improvement in financial instruments is necessary, including the reinforcement of existing ones, the establishment of new financial models, the development of supporting mechanisms, and more active and proactive engagement of financial institutions (Bertoldi et al., 2021. Financial instruments for energy efficiency can take the form of debt or equity financing. In the EU, they typically range from conventional instruments such as subsidized loans to new or emerging models in the European market such as crowdfunding, savings accounts dedicated to refurbishment, and so on. Energy efficiency mortgage (EEM) financing models, which are widely used in the USA (Xu et al., 2009), are becoming increasingly popular in the private sector in Europe (Bertoldi et al., 2021. Furthermore, the latest proposal for the recast Energy Performance of Buildings Directive (European Commission, 2021) has highlighted the importance of EEM in mobilizing the real estate market towards sustainable finance, in line with the European Green Deal.

EEMs provide the possibility of choosing a bank loan that has reduced rates for buying energy-efficient buildings or those that have been energy retrofitted. EEMs can also be used to purchase existing buildings that will undergo energy improvements. In this case, they are called energy improvement mortgages (EIMs). Through EIMs, borrowers can include the cost of energy efficiency improvements in the mortgage without increasing the down payment. In Europe, this new financing model is being promoted as the Energy-efficient Mortgage Action Plan (EeMAP). The main objective of the plan is to create a community-wide energy-efficient mortgage (EEM) market that is available to households and businesses. In 2020, the European Mortgage Federation (EFM) announced that ten banks in Italy are preparing to launch this new type of loan on the market. EEMs have the advantage of lower interest rates or loans with a more favourable value and a variable rate that progressively decreases as energy efficiency increases (EFM, 2019).

Access to finance has long been the main obstacle for European consumers who are considering home ownership (ECSO, 2019). This is true especially for sustainable properties, which tend to have above-average prices. For instance, in recent years, young families in Italy have been strongly penalized from entering the sustainable real estate market. In particular, energy-efficient properties that often have high prices are out of reach for young people who commonly have limited financial resources. In light of this, if properly implemented, EEM can be a viable financial debt tool to help young households enter the real estate market for sustainable buildings. Thus, to enhance the attractiveness of energy-efficient buildings, potential household preferences should be better understood, and the mortgage plan designed appropriately.

Although EEM is a financial product that is already offered by some Italian financial institutions (10 banks, as of 2020), it is too early to observe consumer preferences using revealed preference-based methods due to low uptake and the short period of adoption. For these reasons, a choice experiment (CE) was selected as the study method in this paper to elicit buyers’ preferences in the real estate market (Hensher et al., 2015; Johnston et al., 2017). A CE consists of asking a group of individuals (in this case, potential buyers) to select from a set of choice experiments the alternative of the greatest utility. Then, through a statistical analysis of their preferences and with the help of a discrete choice model, we deduce the marginal utility that supports each characteristic of the evaluated options.

In our study, the CE aims to provide policymakers with the marginal rates of substitution between attributes (Aravena et al., 2014) that determine the choice of high-efficiency properties and an EEM loan. To achieve this, the CE considers attributes of varying nature, such as different flat types by energy class, financing mode (whether through a standard credit plan or EEM) and monthly instalments, to explore the preferences of young, well-educated consumers as potential home buyers.

This study is one of the first to provide information on household preferences for housing and to determine whether EEM can help in the energy transition. Starting to explore the appreciation of EEMs could ensure that credit schemes are designed around the needs and desires of consumers and bring this standardized scheme to consumers across Europe. The target population of this exploratory study consists of a randomly selected sample of young people living in the metropolitan area of Turin (Italy). Although this survey was developed in a limited geographical area, it represents a first pilot case to investigate consumer appreciation of this tool, which is still not very widespread.

The paper is organized as follows. After the “Introduction”, the “Research background” section explores the research in studies on consumer preferences in the housing sector, the issue of housing affordability, tools to support energy investments, and applications of stated preference methods in the energy sector. The “Methodology” section presents the method and working framework. The sampling method, questionnaire structure, and CE experimental design are presented in the “Survey set-up and data collection” section. The results are presented and discussed in the “Results” section. The implications, limitations, and conclusions are summarized in the “Conclusions” section.

## Research background

### Financial tools to support energy investments

Scholars have pointed out that household choices depend on user preferences, household needs, and financial resources (Norris & Shiels, 2007; Squires & Webber, 2019). In addition, macro-level factors, such as the state of the housing market, public policies, and the economic situation, can influence household choices in the same way. These factors are intrinsically linked, as their combination determines inclusion or exclusion from a specific segment of the housing market (Bravi et al., 2010; Kuang & Li, 2012). Factors that affect access to the housing market can be divided into fiscal, regulatory, or monetary terms. Fiscal factors include tax subsidies to allow access to affordable housing, granted by public actors to provide housing or to promote adaptation programmes (Gibb & Whitehead, 2007). Regulatory factors are prepared to control the quality standards that are guaranteed in urban plans to ensure affordable housing (Austin et al., 2014; Norris & Shiels, 2007). Monetary factors are mainly concerned with appropriate interest rates for house purchase and setting constraints for access to credit (Squires & Hutchison, 2014; Zhu et al., 2017). With a focus on this latter issue, the mortgage market has changed considerably in recent years in Italy. After a period of steady expansion that began in the late 1990s, the real estate cycle reversed its trend well before the international crisis of 2008. In 2006, real estate investments and profits entered a downward phase. The duration and intensity of the crisis had a much stronger negative impact on the business system than in other European countries, as Italy is characterized by more fragile economic and financial conditions. For banks, this has meant a drastic reduction in credit, with repercussions on their ability to grant new loans (Panetta & Signoretti, 2010). The inability to obtain credit from banks has particularly affected high-priced investments, such as energy-efficient buildings. In 2002, the energy-efficient renovations of buildings, introduced by the Energy Performance of Buildings Directive (EPBD) and its subsequent recasts until 2021, and processes of long-term renovation strategies initiated an unprecedented path of energy transition of the building stock in EU Member States (European Commission, 2002, 2010, 2018, 2021) and led developers to adapt the real estate market to the new political and environmental rules. As a result, renovations and new energy-efficient buildings are expensive and private owners may not have the means to finance them. To support private investors and home owners/users by addressing financial and investment gaps, several financial instruments have been provided by EU countries (Bertoldi et al., 2021. Polzin et al. (2019) reviewed in depth the policies developed over the past two decades to support the directives and regulations that have been imposed. In general, three families of economic policies can be recognized to support the implementation of renewable energy sources (RESs) and building efficiency measures: direct investments, fiscal and financial instruments, and market-based instruments. Direct investments include financing policies aimed at direct acquisition of the generation capacity of RES by public authorities, such as funds for sub-national governments, infrastructures, supply rules, and funding for research, development, and deployment. The financial and fiscal policies directly affect efficiency interventions, which provide, for example, remuneration derived from the sale of the energy produced (feed-in/premium rates), subsidies, loans, tax relief, and guarantees for private investors. The market-based instruments include the Emission Trading Scheme and green certificates. The greenhouse gas emission allowance trading system introduced by the European Union in 2005 was conceived with the aim of inducing large European companies to pollute less. Green certificates constitute an incentive mechanism for the production of electricity from RESs (Dell’Anna, 2020).

In Italy, following the indications of the measures set out in the Clean Energy for All Europeans package (Commission, 2016), the 2017 National Energy Strategy (NES) confirmed the crucial role of energy efficiency in Italy’s energy transition path, undertaken with Legislative Decree 102/2014 and the Energy Efficiency Action Plan of 2014 (SEN, 2017). In the regulatory sphere, these guidelines have helped to strengthen energy efficiency policy. The policies developed over these years have encouraged Italian households to invest in energy efficiency. In fact, according to ENEA’s eighth energy efficiency report (ENEA, 2019), Italian households invested over €39 billion in energy upgrading projects between 2007 and 2018, including €3.3 billion in 2018. Thus, increasing energy efficiency in buildings is a priority goal for the country. In Italy, the main incentives that are still available to promote energy efficiency in the private residential sector are the tax deductions and the “Conto Termico” (Ministry of Economic Development, 2019). Tax deductions (referred to as “Ecobonus”) were updated by Law No. 205 of 27 December 2017, which extended tax deductions in personal income tax for the energy retrofit of buildings. The deduction rates are differentiated according to the investment made and the economic benefit in terms of the achievable energy savings. The incentives are for interventions in individual property units and common areas of condominiums with different taxation rates. The Italian Ministry of Economic Development recognized the potential of incentives to promote the green economy in the recession during the COVID-19 pandemic and established the “Superbonus 110%” measure in May 2020. This incentive provides a 110% deduction of the expenses incurred for interventions that improve the energy efficiency of buildings and reduce seismic risk in the residential sector. The second tool is “Conto Termico 2.0”, which has been in force since 31 May 2016. It is a public contribution that promotes an increase in energy efficiency and the production of renewable energy. The “Conto Termico 2.0” cannot exceed 65% of the eligible expenditure incurred and is designed for public administrations, individuals, and businesses. The incentives include feed-in tariff and feed-in premium measures. The latest update also introduced the possibility of obtaining an “Ecoprestito” through the issuance of guarantees on loans granted by credit institutions to citizens for the energy upgrading of buildings. The “Ecoprestito” is one of the innovations introduced with “Bonus casa 2019” (2019 House Bonus). With this financial measure, the Italian state is in fact acting as a guarantor for citizens to facilitate loans from banks and credit institutions. This action is limited to energy retrofits on their own or rented properties, at rates below the market average up to a maximum expenditure limit of €96,000.

Alongside the instruments guaranteed by the Italian state, the EEM financial product is spreading in Italy^1^ among banking institutions. In 2018, the board of the European Investment Bank (EIB) approved the creation of a new financial instrument called Smart Finance for Smart Buildings. The aim is to make investments in energy efficiency projects in residential buildings more attractive and accessible to private investors, through the smart use of EU grants as collateral, and to deliver on the commitments of the Paris Agreement adopted by consensus on 12 December 2015. Within this framework, the EeMAP initiative is a clear signal that both the European mortgage and covered bond markets are looking to upgrade the current green mortgage system through EEM. The EeMAP project intends to create a standardized mortgage European-wide energy efficiency loan to encourage the retrofit of buildings and the purchase of highly efficient properties through financial instruments that make the purchase conditions favourable: lower rates for homes that are already efficient, and extra capital to implement energy retrofits in inefficient ones. The EeMAP initiative is based on two key insights: investing in building performance improvements can help loan applicants free up available capital through lower utility bills and it can increase the value of the property (Richardson, 2019). As a result, these investments reduce the risk of mortgage default and thus represent an attractive solution for all stakeholders: lenders, investors, and consumers. In Italy, green home loans finance the purchase of an energy-efficient home (A or B rated only). These loans benefit from favourable conditions such as reduced interest rates. EEMs are offered at fixed and variable rates, with repayment plans of varying duration, depending on the bank. In Italy in October 2021, considering a fixed rate for the entire duration of the loan for purchasing a home, and a loan to value ratio between 50.01 and 70%, at Banca Intesa San Paolo interest rates ranged from 1.15% for a 6-year mortgage to 1.45% for 30 years. The EEM plan envisages a reduction of 10 basis points from the average market rate proposed by Italian banks. Loans are available for the purchase of a new home and for expenses incurred during energy retrofit works (EFM, 2019). Moreover, the EEM does not exclude the possibility of taking advantage of the tax deductions that are available under current legislation, which makes retrofitting more affordable for low-income households.

### Italy’s mortgage market

Italy has always been characterized by a high percentage of property owners compared to other European countries such as Germany, Austria, and France. In 2018, the European average of owners out of the total population was 69.3%, while in Italy the figure stood at 72.4% (Eurostat, 2019). Compared to 2015, there was a decrease in ownership and an increase in the number of households renting residential properties. There has been a reduction in households with mortgages in recent years, in line with policies restricting access to credit. However, national differences can be seen in an analysis of the performance of individual markets by age group and the regional area between 2017 and 2019. An analysis of age groups showed that ownership increased by 5.58% in 2019 to favour those over 65. In contrast, a 2.72% decrease in individuals living in their own homes was recorded for those under 35 in 2019 compared to 2017. Young families were the group that was most affected by the new financial constraints established in 2019 by banking institutions (Agenzia delle Entrate, 2019). This was also confirmed in a study conducted in Italy by the Tecnocasa Group’s Studies Office, which analysed a sample of sales made through its affiliated agencies and found that the average age of homebuyers in Italy has risen (Ufficio Studi del Gruppo Tecnocasa, 2020). The analysis based on sales made in 2019 throughout Italy shows that the age group that was most active in terms of buying and selling on the market was that of 35- to 44-year-olds (27.8%), followed by 18- to 34-year-olds (27.1%). This trend is undoubtedly linked to the fact that young people are now more inclined to rent because of the difficulties they often face in accessing credit and because of their housing choices. It is possible to conclude that the financial crisis that has characterized Italy in recent times and the fluctuation of bank interest rates are strongly influencing the choices of potential property owners in Italy. Income and interest rate fluctuations can generate unsustainable financial choices for consumers if they are not properly considered. Therefore, it may be useful to carefully assess the preferences of consumers between 18 and 44 years old as potential property buyers and to check whether new financial products could make it easier for them to access the real estate market.

### Paper contribution

The question posed by this research is whether a private financial instrument can help households enter the market of sustainable real estate, which is generally characterized by higher market prices. According to the Immobiliare.it research department, the average price of property in Italy is 1940 euros per square metre, while the average cost of a class A home is 2618 euros; a difference of 34% that can reach peaks of 60% for particularly prestigious projects, perhaps in class A +  + or near the city centre (Baiardi, 2018).

Several branches of research have contributed extensively by investigating the decision-making problem of choice in real estate (Cosacov, 2019). In particular, researchers’ attention has increasingly focused on understanding preferences and choices for intrinsic and extrinsic housing attributes (Bottero et al., 2019; Zinas & Jusan, 2012). In econometrics, revealed preference and stated preference models have had great resonance in the field of energy investment decisions. In particular, stated preference is useful when there is a paucity of data from the market (Banfi et al., 2008; Bragolusi & D’Alpaos, 2021; Encinas et al., 2018; Kim et al., 2021; Scarpa & Willis, 2010). While studies on household choices of energy investments and related policies have been widely investigated, few studies have included attributes related to credit and financial plans. In this research area, Zander et al. (2019) conducted a choice experiment (CE) to define Australian households’ preferences of financial incentives for installing residential rooftop photovoltaic panels. The outcomes show that the main driver for the decision to install photovoltaic panels in the future is the installation cost, the longer time required to sell the solar energy, and the solar energy tariff. Jeong (2013) studied how government supports influence users’ preferences for a microgeneration system in Korea. According to the results, a system with low installation costs but high energy-saving benefits and long warranty periods is preferred. Households prefer direct subsidies over low-interest loans. The review shows that no studies have been conducted in Europe on EEM. Above all, no study has focused on analysing the preferences of new property ownership or on analysing a credit plan.

This research differs from previous studies that have investigated household preferences for a hypothetical property using discrete choice methods. Previous studies mainly focused on technological factors that influence home purchase. There is a lack of perspective that credit plans can influence consumer choice, although monetary factors are important in investment choices. Given that the choice experiment has been widely used to assess preferences in the real estate and energy investment sector, the method seems suitable to answer this question. This is especially true as it is too early to implement revealed preference methods due to the lack of sufficient data. The study contributes to the existing literature by initially investigating consumer attitudes to energy and environmental sustainability. An analysis of the sample’s attitudes is used to understand whether the respondents are aligned with those of other studies conducted in Italy and to validate the answers accordingly (“Energy efficiency perception” section). Secondly, the research investigates through econometric analysis whether the new EEM financial product provided by private banking institutions can be used by consumers to purchase energy-efficient properties or existing properties that have undergone an energy retrofit (“Respondents’ energy efficiency and financial knowledge” section). In particular, the study investigates through a CE-based survey whether EEM is a product that can replace or complement other government support in Italy. Given that the literature has shown that young people have a high level of adherence to the topic of sustainability and given that analyses conducted in Italy have revealed that the threshold of potential homebuyers has risen to individuals aged 44 years, we carried out an exploratory survey of all potential age groups for the purchase of a new home (18–44 years).

## Methodology

### Choice experiment model

Energy consumption, investments in energy efficiency, and pro-environmental actions involve individual decision-making and behaviour. However, behavioural economics and related literature shows that individuals do not always choose the outcome that maximises their well-being (Frederiks et al., 2015). Therefore, policymakers should intervene and attempt to induce welfare-maximising outcomes by providing appropriate default options and establishing an appropriate choice set (Dell’Ovo et al., 2020). This study uses the stated preferences methodology, more precisely CE, to analyse individual housing preferences. The CE approach is based on microeconomic choice theory and random utility theory (RUT). It argues that each individual has a trade-off of preferences among possible choice alternatives, which satisfies the axiom of rationality. According to this principle, consumers make their choices rationally, considering the technological and economic situation, and personal needs (Mas-Colell et al., 1995).

The individual evaluates the usefulness of an alternative according to Eq. (1):

1 $${\mathrm U}_{\mathrm{ij}}={\mathrm V}_{\mathrm{ij}}+{\mathrm\varepsilon}_{\mathrm{ij}}$$

where ${U}_{ij}$ is the utility of choice of the $j$-th product, among the $M$ alternatives available $(j=1, \dots , M)$, ${V}_{ij}$ is the observed utility, and ${\varepsilon }_{ij}$ is the error term. We assume that the individual $i$-th chooses the $j$-th alternative on the $y$-th, so if ${U}_{ij}>{U}_{iy}$, we can define the probability as (2):

2 $$\mathrm P\left({\mathrm x}_{\mathrm{ij}}\vert{\mathrm s}_{\mathrm i},\mathrm A\right)=\mathrm P\left[\mathrm\varepsilon\left(\mathrm s,{\mathrm x}_{\mathrm j}\right)<\mathrm V\left(\mathrm s,{\mathrm x}_{\mathrm i}\right)-\mathrm V\left(\mathrm s,{\mathrm x}_{\mathrm j}\right)+\mathrm\varepsilon\left(\mathrm s,{\mathrm x}_{\mathrm i}\right)\right]$$

Equation (2) states that the probability of choosing alternative ${x}_{i}$ is equal to the probability that the stochastic part of alternative $j$-th is less than the utility of the alternative $i$-th, minus the observable part of the alternative $j$-th (Louviere et al., 2000). Equation (2) defines the random utility model (RUM). McFadden (1974) linked the theoretical RUM to the statistical discrete choice model, with a specification that can be resolved in the multinomial logit (MNL) model (or conditional logit [CL] model) as follows (3):

3 $${\mathrm P}_{\mathrm j}=\frac1{\sum_{\mathrm i=1}^{\mathrm J}\mathrm e^{-\left({\mathrm V}_{\mathrm i}-{\mathrm V}_{\mathrm j}\right)}}$$

${V}_{j}$ will be a function of the exogenous variables in the model (product attributes), the socio-demographic variables (*s*), and the effects that each variable has on utility. This could be expressed as follows (4):

4 $${\mathrm V}_{\mathrm j}={\textstyle\sum_{\mathrm k=1\;}^{\mathrm K}}{\mathrm\beta}_{\mathrm{jk}}{\mathrm s}_{\mathrm{jk}}$$

where ${s}_{jk}$ is the vector of the exogenous variables (including the socio-demographic variables and the choice attributes) and ${\beta }_{jk}$ is the vector of the parameters (coefficients) linked with each of the attributes of the $j$-th alternative. ${\beta }_{jk}$ can be interpreted as a variation of the dependent variable ${V}_{j}$, in correspondence with each unitary variation of the independent variables ${s}_{jk}$.

The estimation of part-worth utilities associated with each attribute level occurs through advanced statistical techniques such as the conditional logit (CL) model. The CE method has gained popularity in recent years, considering the real choice process adopted by the consumer, and the possibility of estimating the interaction effects between attributes. Indeed, the process implemented by consumers does not include the ranking of all alternatives of choice, as envisaged in traditional conjoint analysis. Instead, the interviewee makes his/her choice indicating the preferred product profile from a limited number of alternatives that coincide with the set of choices available (choice set).

The general goal of this study was to examine consumers’ preferences when they are buying a home. In particular, the property attributes that were considered were the Energy Performance Certificate (EPC) rating, the monthly mortgage payment, the type of loan, the level of maintenance, and the possible hiring of an expert for the energy retrofit. Of particular interest was whether a favourable mortgage for efficient buildings matched the buyers’ choices, considering the house characteristics. In other words, this study estimated the relative importance of the characteristics of the EEM, starting from consumers’ choices. The CE method seemed ideal for this study. Respondents were asked to state their preference for a known private good and had experience with the investigated topic, which limited errors in choice behaviour (LaRiviere et al., 2014). The mortgage payment was chosen as the payment vehicle with which respondents were confident and thus the experiments were realistic. Individuals were carefully selected to ensure suitable experience and appropriate demographic data (Marmolejo-Duarte & Bravi, 2017). As stated before, the target population was randomly selected from young people living in the metropolitan area of Turin.

### Workflow

**Fig. 1:**
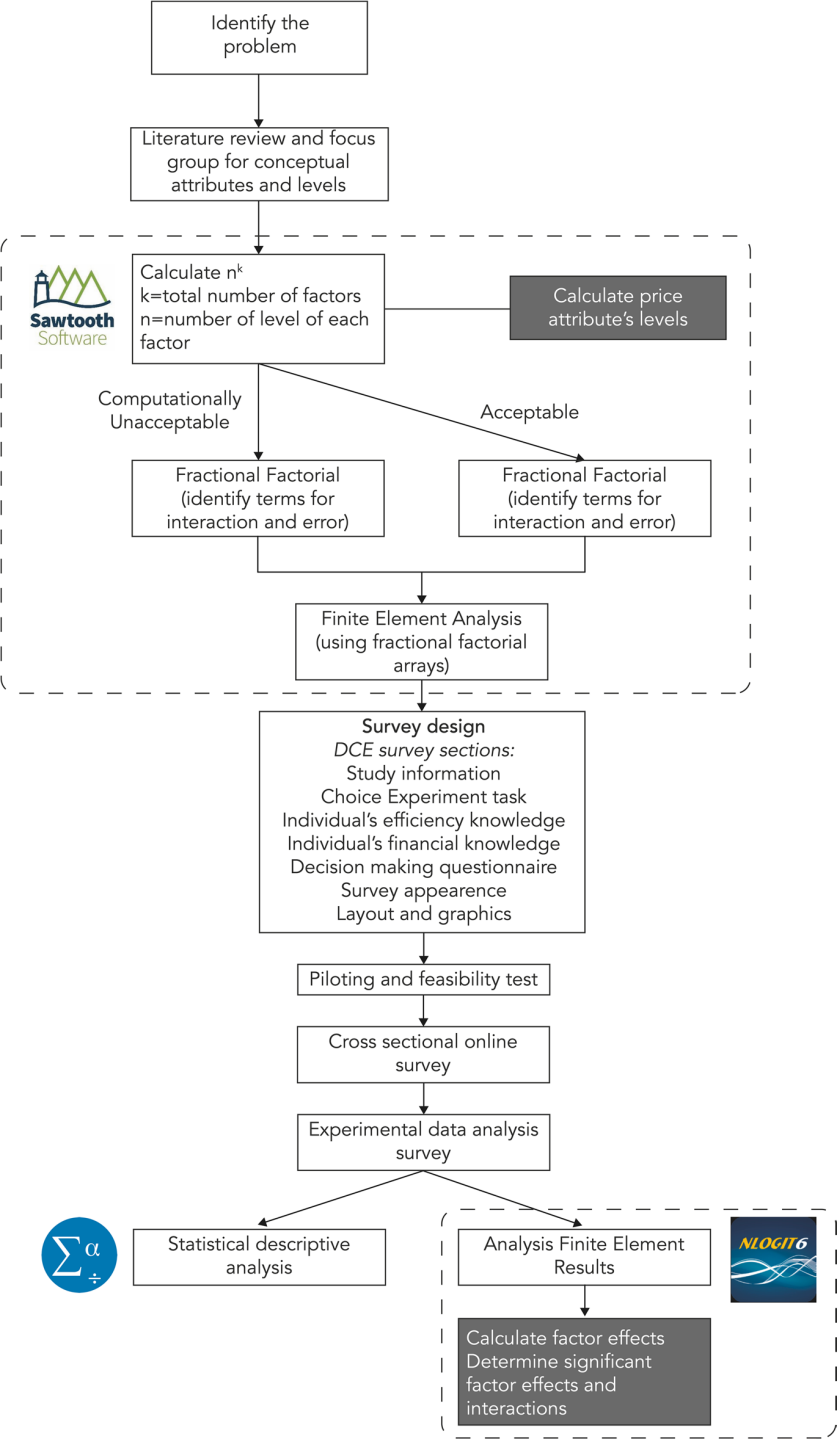
Choice experiment (CE) workflow

The CE approach requires a series of steps linked to each other. An experimental work plan is defined in Fig. 1. The main steps can be summarized as follows.

1. Definition of the objective of the choice experiment, according to the problem to be analysed.
2. Definition of product attributes and selection of levels from the literature review and focus groups with experts.
3. Definition of an experimental plan and related profiles (choice set) to be submitted to the interviewee. The attribute levels were combined to form the alternatives chosen by the respondent. Once the possible combinations of attributes and relative levels had been defined through the fractional factorial design, the next step involved the construction of the choice sets. Sawtooth software was used to randomly combine the alternatives following the constraints imposed by the analyst.
4. Validation of the questionnaire through pre-tests.
5. Selection of a random sample of consumers to be involved in the survey and administration of the survey.
6. Choice of the model for estimation of unknown parameters.
7. Descriptive analysis.
8. Econometric analysis

## Survey set-up and data collection

### Sampling method and data collection

The lower age limit for participating in the survey was set at 18. The limit for age groups that is generally used in statistical studies on mortgage applications and lending in Italy (Agenzia delle Entrate, 2019) was maintained. The questionnaire was aimed at residents of the Metropolitan City of Turin. Northern Italy is closer to the average European socio-economic and climatic conditions. Furthermore, most residential buildings in Italy are located in the average climate zone E (47.4%), including Turin and its metropolitan area (Ballarini et al., 2014; ISTAT, 2011). Moreover, Turin is one of the most commonly studied cases of EPC in Italy (Bottero et al., 2018; Dell’Anna, Bravi, et al., 2019; Dell’Anna et al., 2019a, b; Fregonara et al., 2014, 2017), so it seems logical to relate this study to the conclusions drawn from existing research. This was an advantage but also a limitation, as the conclusions cannot be extended to all of Italy and other northern regions. However, the selection of a sample in a given geographical area may be interesting for a preliminary exploratory survey.

The interviews were conducted from March 2019 to June 2019, through an internet-based questionnaire on the Sawtooth Software Market Research Tools (SMRT©) platform. The questionnaires were administered online and in computer-assisted, face-to-face interviews. This combination of modalities compensated for the biases that characterise survey methods. Face-to-face interviews may give rise to complacency bias, but they allow clarification of questions that may be difficult for some individuals to understand and they provide access to non-digital users. At the same time, online surveys allow respondents to freely express their opinion. There were no systematic differences between the two interview methods. In the face-to-face interviews, the respondents were not questioned orally by the research assistant. The respondents were free to answer the questions using the computer to avoid complacency bias. In this way, differences in the interviewing method were reduced.

To obtain a consistent sample, we eliminated CEs that took less than 5 min or more than 25. In the first case, a careful answer set is unlikely to be produced in under 5 min. In the second case, it is highly likely that the questions or CEs were not understood. In addition, we eliminated responses from the same internet protocol (IP) address to avoid overrepresentation of individuals from the same household. In total, the analysis consisted of 820 valid choice tasks from 205 respondents. No statistical differences were observed in responses between e-mail questionnaires and those obtained in person.

### Questionnaire

The questionnaire consisted of four sections. The survey was introduced by a brief presentation of the objectives pursued by the research. This should have convinced consumers to participate, and reassured them about the usefulness and purpose of the information provided. The English translation of the questionnaire is attached in Appendix 1.

The four sections were as follows.

1. The first section began with the presentation of alternatives that can characterize a multifamily home. The attributes selected for this study are the type of property (located in a new, retrofitted, or to be retrofitted building), the hiring of a technician to guide energy restructuring, the type of mortgage (standard or green), and the discount on waste tax (TARI, TAssa RIfiuti). The latter attribute was added to test whether people might have more confidence in subsidies provided by a public institution (such as the garbage tax rebate) or in EEM-based private funding. The monthly mortgage payment resulting from the combination of features was included in each alternative as an informational attribute only. Since a target population living in Turin was selected to develop the exploratory survey, the price attribute levels were defined from the results of a study based on the hedonic price method developed in the metropolitan area under study (Dell’Anna, Bravi, et al., 2019; Dell’Anna, Vergerio, et al., 2019). Subsequently, each interviewee was presented with four choice sets with three profiles each.
2. In the second section, questions were asked about the significance of certain energy efficiency benefits to the consumer, with responses on a 4-point Likert scale (from not important to very important). The benefits referred to savings on electricity and gas bills, possible reduction of the waste tax, a reduction in bank interest, an increase in the value of properties, improvement of internal comfort and health, and a reduction in environmental impacts (Crespo Sánchez et al., 2021). In addition to the benefits, the disadvantages generated by housing renovations were evaluated (delayed entry into the new home, inconvenience, and stress). Furthermore, data were requested on the current property in which the interviewee resides, and their priorities for renovation, from an energy, aesthetic, and internal distribution point of view (Becchio et al., 2018; Bragolusi & D’Alpaos, 2021). Information was also requested on the sustainable actions that the interviewee usually does and on what, in their opinion, affects the energy bill (Buso et al., 2017; Crespo Sánchez et al., 2021).
3. The third section explored the respondent’s financial knowledge, their assessment of investment risks, and personal opinions on the advantage of the type of rate used in mortgages.
4. In the last section, the socio-demographic profile of the interviewee was traced by asking questions about age, gender, income, and family unit. These data were very useful for testing the quality of the interviewed sample and validating the collected data.

### Experimental design

As explained before, four attributes with different levels were identified for this experiment (see Table 1). They were designed to describe the characteristics that guide the consumer in choosing a home.

**Table 1:** Attributes and levels of the choice experiment

Attributes	Levels	Variable name
Type of dwelling	A-rated apartment that does not require an energy retrofit	A
	E-rated apartment retrofitted to C-rated with the help of an energy expert	ECS
	E-rated apartment retrofitted to C-rated without the help of an energy expert	EC
	E-rated apartment	E
Type of loan	Standard mortgage	S
	Energy-efficiency mortgage	EEM
Waste tax discount	0% discount on waste tax	WT0
	10% discount on waste tax	WT10
	20% discount on waste tax	WT20
Price	Monthly payment	P

The first attribute identified the “type of property” and was divided into four levels. The differences in level were mainly related to energy performance. The energy class was used to define the levels based on the Energy Performance Certificate (EPC) introduced by the Energy Performance of Buildings Directive (EPBD) 2002 (European Commission, 2002), which has already been revised twice (Economidou et al., 2020). The attribute of energy class referring to EPC was already used extensively in similar studies as it is a very common sustainability indicator with which respondents are confident (Lee et al., 2018; Marmolejo-Duarte & Bravi, 2017). The energy label is not only applied to buildings, but also to electrical appliances commonly found in homes (Skourtos et al., 2021). The attribute included four possible conditions of the apartment. The first level was a class A flat that required no energy retrofit. The second level was a class E flat, retrofitted to class C with the assistance of an energy expert. The third level was a C-rated flat retrofitted without the assistance of an expert to supervise the work. The fourth level was an E-rated flat. As mentioned above, the attribute also referred to the technical assistance of an energy expert for the retrofitting of the dwellings, who carried out an energy audit, planned the retrofit, recruited suppliers, obtained permits, and supervised the work. The attribute was not applicable in the case of A- or E-rated flats.

The second attribute referred to the “type of loan” for the payment of 80% of the value of the home. The attribute had two levels: the standard loan promoted by Italian banks for the purchase of a primary home, and the EEM loan. The latter could be used for the purchase and retrofit of the apartment (if desired). The financing was similar to traditional mortgages, but with lower interest rates for all apartments rated C or higher. In this case, the rate varied according to the rating of the apartment purchased.

The third attribute considered a discount on the waste tax (WT) imposed on the dwelling. It was intended to introduce an attribute on municipal spending in the choice experiment, to test respondents’ confidence in financial instruments of a public nature. Moreover, it was interesting to see whether a discount for a public service could be a driver for energy efficiency. As waste tax is an annual payment determined by the characteristics of the house, respondents should be able to compare it with other expenses during the year that are also linked to the characteristics of the house, such as energy efficiency or mortgage payments.

An important part of defining choice alternatives was to provide comprehensible information on which respondents could base their perceptions of the marginal utility of the levels and attributes. As stated by Huber and Zwerina (1996), a lack of information on the utility of attributes limits the application of CE (Lakić et al., 2021; Mangham et al., 2009). Consequently, the alternatives were accompanied by an informative price attribute. In this case, due to the nature of the case study analysed, a monthly payment was assumed. The attribute was considered the loan instalment, to which was added the cost of rehabilitation (if any) and the energy expert’s fee. The energy savings and WT discount were subtracted, depending on the composition of the chosen tab. The length of the loan was established on the basis of the average duration in Italy of 20 years. The total price was defined as the marginal cost of the characteristics of the property plus the additional cost of attribute two minus any benefits provided by attributes three and four. The resulting figure was divided by the number of expected instalments (Dell’Anna et al., 2019a, b). The attribute was considered the mortgage payment, adding the cost of rehabilitation (if any) and the energy expert’s fee, and subtracting the energy savings and the WT discount according to the composition of the choice set card.

## Results

### Sample description

Before the questionnaire was administered, approximately 25 interviews were conducted as a pre-test. Experts from the real estate and energy sectors were primarily involved. Since the questionnaire was aimed at consumers, non-specialists were also asked to complete the questionnaire. Once everyone had completed the questionnaire and provided comments and suggestions, the final version was widely distributed.

We received a total of 462 questionnaires. A total of 244 questionnaires were fully completed. As the aim of the study was to analyse the preferences of young potential home buyers, the sample was restricted to this consumer group. To define the final sample to be used for the development of the descriptive and econometric analysis, the sample was restricted to respondents aged between 18 and 44. Although respondents under 20 are characterized by a low potential to purchase property, we referred to the ranges commonly used in studies of consumer demand for mortgages (Agenzia delle Entrate, 2019), which also include the population in this age group. We only considered completed questionnaires of respondents aged between 18 and 44. Consequently, 205 interviews were used for the analysis. All participants were residents in the metropolitan area of Turin. As shown in Table B1(Appendix 2), the sample of respondents consisted of people aged between 18 and 44 years: 31.7% between 18 and 24 years, 41.5% between 25 and 34 years, and 26.8% between 35 and 44 years. There was an equal distribution of men and women: 54.6% and 45.4% respectively. Half of the sample (50.2%) had obtained a university degree and 56.6% of the respondents were workers. About 40% of the respondents stated that they received a net monthly household income higher than the average income in the northwest of Italy (€2886/month in 2019) (ISTAT, 2020).

### Energy efficiency perception

**Fig. 2:**
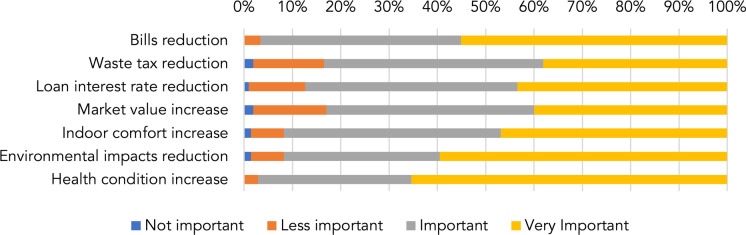
Perceived benefit and co-benefit of energy efficiency expressed in %

From the attitude questions, respondents were aware of the benefits and co-benefits of energy efficiency (Fig. 2). Among the most important perceived benefits were reduced energy bills and increased indoor comfort and health conditions in homes. Respondents seemed to be sensitive to the environmental effects of lower CO_2_ emissions. A possible reduction in waste tax was considered less important than the other attributes. Interestingly, the perception of an increase in health was considered to be as relevant as energy savings (Table B2, Appendix 2).

**Fig. 3:**
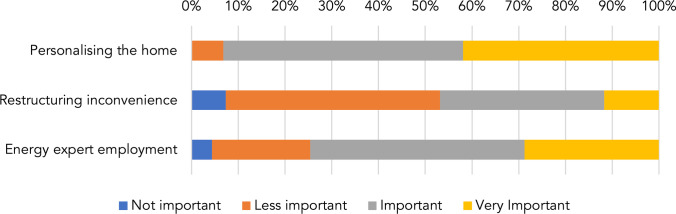
Opportunities and threats of energy retrofitting expressed in %

Respondents saw energy retrofits as an opportunity to personalise their homes (Fig. 3). A total of 93.2% responded that the opportunity for personalization was important or very important. Discomfort due to energy retrofits was not an area of concern for respondents, as only 11.7% stated that it is a very important consequence to consider. The energy expert is an important professional figure to involve in home retrofitting. The results show that the renovation of existing building stock was seen as a potential opportunity rather than a threat. This should lead policymakers to think about incentive policies and incentives for the renovation of building stock rather than for the construction of new buildings.

As for trust in institutions to promote retrofit operations, half of the sample reported low trust in measures of a public nature (56.6%). The lack of confidence can be determined by the fact that the measures taken by governments to promote private investment in energy efficiency are considered insufficient or difficult to achieve (Boriani et al., 2020). As for private institutions, such as banks and financial institutions, the percentage of those who believe in them was slightly lower (51.2%). The low level of confidence may be driven by the limited availability of financial instruments to support energy retrofitting from these institutions or a lack of knowledge about them. A third reason could be financial barriers defined by the restrictions imposed by institutions.

**Fig. 4:**
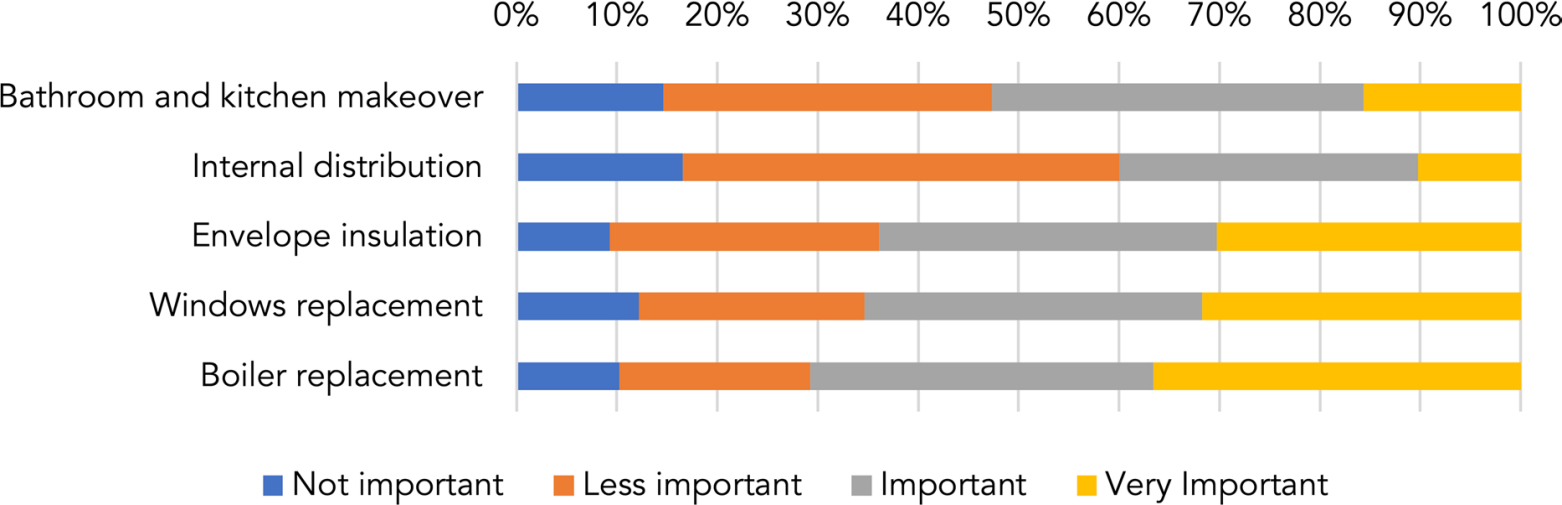
Retrofit priorities in interviewees’ homes expressed as a %

From the questions about their own home, the interviewees’ asset profiles were obtained, and the intervention priorities for housing renovation. A total of 31.2% of the respondents were living in their own house, and 14.6% were paying a mortgage. A total of 29.3% of the sample were paying rent (Table B3, Appendix 2). The rest of the respondents were living in a property without paying rent or a mortgage. Most of the respondents stated that the house they were living in at the time of the survey needed energy retrofitting in terms of insulation of the envelope and replacement of the boiler (Fig. 4). This was followed by bathroom or kitchen renovation. According to the results, the living conditions in the houses are worrying as most of the respondents emphasized the urgent need for an energy retrofit rather than an aesthetic one. This conclusion was particularly true in the case of wall and window insulation, which are the main elements of the building envelope. This result is in line with ENEA (Agenzia nazionale per le nuove tecnologie, l’energia e lo sviluppo economico sostenibile) reports that describe the Italian building stock as inadequate. According to this report, about 13 billion square metres of housing require renovation (ENEA, 2019). This result is partially related to the fact that most of the homes in Italy are located in multi-family properties whose energy improvement (energy system and envelope) requires the consent of the majority of owners, which makes it difficult to retrofit the buildings.

In terms of the interviewed individuals attitudes, most of them separated their waste, purchased efficient appliances, and tried to minimise consumption in their own homes (Table B4 Appendix 2).

### Respondents’ energy efficiency and financial knowledge

From the energy knowledge of the respondents, the building characteristics (opaque and transparent envelope), systems for heating and cooling, and user habits were the main factors that influence energy bills (Table B5, Appendix 2). Respondents were aware that building characteristics can have an impact on the level of consumption. In terms of financial knowledge, investing in government bonds was considered less risky than buying shares on the stock exchange. However, many respondents did not know how to answer the question (49.8%). Half of the respondents did not know whether the rates of a fixed-rate mortgage are higher than a variable-rate mortgage. Most respondents believed that a profitable investment requires a higher risk (56.1%).

### Econometric analysis

The data collected in the CE were analysed using the McFadden (1974) conditional logit (CL) model. The objective of the study was not to provide a monetary value for the attributes but rather to obtain respondents’ estimations of the relative value of the attributes of a potential house to buy in terms of marginal rates of substitution. Thus, the variable coefficients estimated by CL can be used to estimate how respondents evaluate different attributes and analyse their trade-offs. The importance of each attribute and each level was estimated using the NLOGIT v.6 module (Greene, 2000). The dummy-variable coding method was used to analyse the data, transforming the categorical variable into a series of dichotomous variables (variables that can have a value of zero or one only) (Hauber et al., 2016). For all levels of the categorical variable except one, a new variable will be created that has a value of one for each observation at that level and zero for all others. The level of the categorical variable that was coded as zero in all new variables represented the reference level. For example, in the case of the attribute “type of dwelling”, the category E flat was the reference level and was omitted. For the attribute “type of loan”, the standard level (S) was taken as reference. For the attribute TARI, the levels were considered an ordinal variable, where 1 indicated the 10% discount level, 2 the 15% discount level, and 3 the 20% discount level. PRICE expressed the monthly payment in euros, taking into account the mortgage payment adjusted for any extra costs incurred for the retrofit, and the economic benefits to be gained.

Direct inspection of Table 2 shows that most of the estimated coefficients were statistically significant at the 5% level. EEM and PRICE were not significant. From this first model, the A-rated apartment was the most popular (β_A_ = 1.88544). This was followed by the ECS (C-rated dwelling retrofitted with the help of the energy expert) and E (coefficient *β* equal to 1.54447 and 0.75592 respectively) types. The waste tax rebate was appreciated by the respondents, although with a lower utility magnitude.

**Table 2:** CL model results

Variables	*β*	SE		*z*		Prob	95% confidence interval	
A	1.88544***	0.31371		6.01		0.0000	1.27058	2.50030
ECS	1.54447***	0.18280		8.45		0.0000	1.18619	1.90276
EC	0.75592***	0.19731		3.83		0.0001	0.36920	1.14264
EEM	0.76384	0.52312		1.46		0.1442	− 0.26146	1.78914
WT	0.03774***	0.00683		5.53		0.0000	0.02436	0.05112
P	− 0.00520	0.00420		− 1.24		0.2159	− 0.01344	− 0.00304
A_1	0.18881*	0.10696		1.77		0.0775	− 0.02082	0.39844
A_2	0.24675**	0.10777		2.29		0.0220	0.03553	0.45797
*Model fit*								
Number of observations			820					
Number of respondents			205					
Adjusted pseudo *R*-squared			0.3107					
Log-likelihood function			− 896.4179					
Inf.CR.AIC			1245.9					
AIC/N			1.519					

*SE*, standard error; *A*, A-rated dwelling; *ECS*, C-rated dwelling retrofitted with the help of the energy expert; *EC*, retrofitted C-rated dwelling; *EEM*, energy-efficiency mortgage; *WT*, waste tax discount; *P*, price: *A_1*, alternative 1 constant; *A_2*, alternative 2 constant

***, **, *Significance at 1%, 5%, and 10% level

As two variables were not significant, we checked whether the inclusion of socio-economic variables would improve the performance of the model. As stated by Hanemann (1984), the structure of the utility function consisted of an observable deterministic element and a stochastic element representing the unobservable component of individual choice (Eq. 1). The inclusion of socio-economic factors can reduce the causal component of the error, which often includes unobserved attributes, unobserved tastes and preferences, measurement errors, and the use of instrumental variables (Ben-Akiva & Lerman, 1985). Therefore, the authors thought to include within the model a variable that considered the economic availability of the respondents. Socio-economic variables (such as income and age) must be introduced through interactions with the alternative specific constant (ASC) or attributes, as they are invariant across choice sets (Bennett, 1999; Greene, 1997). They would cause a Hessian singularity. Introducing the ratio of net monthly income to price (mortgage/income ratio: MIR) into the econometric model overcomes this problem. Specifically, the model assumed that the final accessibility can constitute a constraint in consumer choices. To consider the influence on the choices of the sample that was examined, it was decided to include a specific index. Appropriate measures of accessibility are the MIR or monthly rent/income (RIR), residual disposable net income of costs, and total housing costs (Bravi et al., 2010; Stone, 2006). In this study, to consider the financial resources of the respondents, the MIR was selected. This allowed verification of real cases of families that have opted for a certain mortgage for the purchase of a new home and how it affects the consumption opportunities of other goods and overall level of well-being. In this way, the MIR was calculated by comparing the monthly net income of the family expressed by each respondent with the monthly payment of the loan proposed in the alternative chosen in the choice set. Since socio-economic characteristics are constant across alternatives, they cannot be directly added to the model. MIR helped to consider them, measuring the interaction of income with the price attribute. Table 3 shows the output provided by the analysis of the second model.

**Table 3:** CL model results (including the mortgage/income ratio)

Variables	*β*	SE	*z*	Prob	95% confidence interval	
A	2.07826***	0.19748	10.51	0.0000	1.69121	2.46531
ECS	1.60372***	0.17196	9.33	0.0000	1.26669	1.94075
EC	0.82559***	0.17658	4.68	0.0000	0.47950	1.17168
EEM	1.12376***	0.17012	6.61	0.0000	0.79034	1.45718
WT	0.03763***	0.00615	5.51	0.0000	0.02424	0.05101
MIR	− 3.76287**	1.90814	− 1.97	0.0486	− 7.50275	− 0.02298
A_1	0.19191*	0.10687	1.80	0.0725	− 0.01755	0.40137
A_2	0.24845**	0.10796	2.30	0.0214	0.03686	0.46004
*Model fit*						
Number of observations		820				
Number of respondents		205				
Adjusted pseudo *R*-squared		0.3123				
Log-likelihood function		− 896.4179				
Inf.CR.AIC		1242.9				
AIC/N		1.516				

*SE*, standard error; *A*, A-rated dwelling; *ECS*, C-rated dwelling retrofitted with the help of the energy expert; *EC*, retrofitted C-rated dwelling; *EEM*, energy efficiency mortgage; *WT*, waste tax discount; *MIR*, mortgage/income ratio; *A_1*, alternative 1 constant; *A_2*, alternative 2 constant

***, **, *Significance at 1%, 5%, and 10% level

All the estimated parameters were statistically significant at a significance level of 0.05 and presented signs and values in line with expected ones. The results show that the MIR variable was the most important attribute. The negative sign of the MIR attribute (β_MIR _=  3.76287) meant that if the MIR increases, the likelihood of a high-priced alternative being chosen decreases. The negative MIR value indicates that those with fewer financial resources (high MIR values) limit their preference for higher investments. This result can be seen as a sign of the internal coherence of the preferences expressed by the interviewees. As regards the type of dwelling, options A, ECS, and EC had a positive sign. Therefore, there was an individual predisposition on the part of the interviewees to choose an efficient dwelling compared to the E-rated one. This preference of interviewees decreased with the increase in expected energy costs (β_A_ = 2.07826, β_ECS_ = 1.60372, β_EC_ = 0.82559). Respondents recognized the importance of relying on an energy expert, as they put ECS in the second position among property-type attributes. The EEM coefficient had a positive value equal to 1.12376. The interviewees demonstrated a propensity to choose the new financial instrument proposed by private institutions. They recognized the benefits deriving from the adoption of loans for efficient properties compared to regular loans. The discount on the TARI was positively recognized by the interviewees although with a lower incidence than the other attributes. As this tax has little impact on households’ annual expenditure, respondents gave much more importance to the energy characteristics of the property.

### Discussion of the results

Starting from the hypothesis that in bank mortgage markets the choice of credit plan is an attribute that influences choice and monthly payment, and represents a trade-off with other building characteristics, a CE was implemented in the metropolitan area of Turin (Italy). The role of the EEM was investigated as a tool to help young people enter the sustainable housing market. From this analysis, some methodological and empirical conclusions can be drawn. From a methodological perspective, stated preference modelling related to consumer choice solved the problem of the impossibility of observing the phenomenon from real or surrogate markets due to lack of data.

The exploratory research presented in this paper provided valuable information, although limited to the Turin area, on the attractiveness, relevance, and understandability of EEM. It highlighted the preferences of consumers in the area. From an empirical perspective, the EEM seems to have an important influence on the choice of a city flat, especially when the flat has high energy performance. There are several possible reasons for the preference of EEM over a regular loan. However, this study can only demonstrate these reasons to a limited extent. Our results indicate that benefits provided by sustainable buildings are recognized by respondents and are important in their choice. EEMs benefit from favourable conditions such as a reduction in the interest rates that are applied, and a more favourable loan to value (LTV) ratio. In Italy, the main credit institutions offer green mortgages with better conditions such as lower interest rates, discounts on the bank spread, and specific discounts, for example on insurance policies, for a certain period. This study does not allow the full benefits of EEM implementation to be considered. However, the advantages of the green mortgage over the traditional mortgage are not simply limited to banking advantages. Borrowers have access to the market for sustainable properties, which allows them to benefit from significant savings over the years on their electricity and gas bills. In addition, efficient buildings are generally characterized by higher market prices that are unlikely to decrease in value over time. In this case, green buildings retain their value and reduce the risk of obsolescence. The interviewees, as possible borrowers, were aware that they could take advantage of lower interest rates, more favourable loan to value (LTV) ratios, and significant savings over the years on electricity and gas utilities. The advantage for the bank is that the higher the energy class of the buildings, the lower the risk of insolvency because families have more liquidity if they save on bills, and because a high-efficiency property acquires value over time. Furthermore, as mortgages account for around one-third of the assets of the European banking sector, banks and credit institutions have a clear interest in promoting financial products in line with the needs of consumers and European regulations to increase demand (Della Valle & Bertoldi, 2022; Pelizzon & Riedel, 2017; Solà et al., 2021). This is particularly true in the conditions of an economic recession, such as that experienced in the COVID-19 pandemic, which led to the collapse of sales and a reduction in requests for loans.

Our results are in line with the recent study “Roll out across EU markets”, published by EEMI (Energy Efficient Mortgages Initiative, 2021), which showed strong demand for energy-efficient mortgages. After in-depth interviews that presented the EEM concept to consumer groups in a number of EU countries, a very positive reception was received in Italy, Sweden, and the UK. Against this background, Italy was therefore recommended as one of the most promising European countries for the pilot scheme. Respondents appreciated the option of a home energy efficiency renovation fully managed by a third party, but they also wanted to be able to pursue a more personalized “do-it-yourself” approach, in line with our study (Biere-Arenas et al., 2021). Moreover, the CE results are in agreement with other research conducted using revealed preference-based models in the city of Turin (Bottero et al., 2018; Fregonara et al., 2014, 2017; Mangialardo et al., 2018). The results showed that the real estate market appreciates buildings with high energy classes, with an average increase in value of about 7% for each class change on the EPC scale (Dell’Anna, Bravi, et al., 2019; Dell’Anna et al., 2019a, b). The market rewards buildings with high energy performance, and recognizes sustainability as a critical element in guiding investment choices. Energy class affects the valuations of assets and the timing of their marketing. In this perspective, EEM could lead to a significant improvement in the time to market of energy-efficient properties. The combined effect of higher prices and faster time to market would lead to an increased return on investment for the developer, and economic returns over time for the property owners.

The CE approach seems to offer great potential to inform public and private decision-makers about consumer preferences in the real estate sector. If preferences do not align with current measures, the results will provide an opportunity to develop strategies to improve the affordability of sustainable buildings. This helps to understand whether EEM is a valid tool to speed up the green transition in the building sector. However, several limitations must be acknowledged. One limitation is related to the composition of the sample, which was characterized by a large share of college graduates. In general, the educational level of individuals is related to prior awareness and knowledge of building energy performance (Marmolejo-Duarte & Bravi, 2017) or higher income availability (Becchio et al., 2018). This may lead to incorrect inference of the results obtained from the study. Moreover, the questionnaire did not focus on the type of property in which the respondent lives. There was no distinction between whether the respondent was already living in a multifamily building or in a detached house. Given that multifamily buildings characterize the area under study, we can assume that most respondents live in that building type. However, the building type may represent a limitation as it may affect the effectiveness and applicability of EEM. In the case of new construction, the problem does not arise because retrofitting is not necessary. However, in the case of condominium buildings that require energy efficiency upgrades, it is not easy to apply the EEM. In fact, these buildings often require an agreement between the various condominiums and owners of the building units. However, given the interest shown even in retrofitted housing by the sample of respondents analysed, the results of the survey suggest the need for ad hoc financing measures that encourage the energy transition perhaps by adopting non-invasive measures, such as individual installation, replacement of windows and doors, and ensuring limited energy class improvements. A future study may look at different real estate sectors to investigate how EEM can be implemented. In addition, there are methodological limitations that are common to all CE. One of the main challenges is to select attributes and levels that sufficiently answer the research question. However, our choice of using four attributes and the limited number of four choice sets places fewer cognitive demands on the respondents. Our CE was conducted on a small, homogeneous sample of people living in the metropolitan city of Turin who uses social media/email. The results cannot be generalized to different Italian contexts. However, the study has interesting results that can be compared with what has been learned from similar studies conducted in this geographical area.

## Conclusions

The Paris Agreement aims at net zero CO_2_ emissions by the mid-century. In its recent proposal for a 2050 climate and energy strategy, the European Commission has indicated the need for more intensive actions to substantially improve the energy performance of buildings. In addition, the challenges currently facing the EU in the energy sector have long included growing import dependency, limited diversification, and high and volatile energy prices, which were highlighted most notably by the geopolitical events of early 2022 that threatened the security of some European countries. In order to achieve the goals that have been set, the European challenge is to increase the pace and depth of energy renovations in private buildings. Financing the upfront costs of energy refurbishments is the biggest barrier inhibiting investment in energy retrofitting. Despite various public policies that are implemented to address some of these barriers, investment in buildings remains at sub-optimal levels. To stimulate private investment, the EeMAP project proposed the creation of a European energy efficiency mortgage (EEM). In particular, debt financing in the form of loans was implemented in banks’ financial products to increase investments in energy efficiency through increased liquidity and direct access to capital. EEM has been adopted in several EU countries, including Italy.

Understanding key drivers of consumer demand, along with a deep appreciation of what consumers perceive as valuable, is a cornerstone in designing a marketable financial product. The study uses a choice experiment to elicit stated preferences for the financial incentive for energy-efficient buildings and finds willingness to adopt it in the sample investigated in Italy (Turin metropolitan area). The surveyed sample consisted of young people and families (aged 18 to 44) as potential property buyers. Our results revealed that individuals were willing to pay higher prices for high-energy class properties (A-rated properties according to the EPC) or retrofitted properties (C-rated). Consumer preferences in this regard appeared to be aligned with recent studies conducted in the area using revealed preferences approaches (Bottero et al., 2018; Dell’Anna, Bravi, et al., 2019; Dell’Anna, Vergerio, et al., 2019; Fregonara et al., 2017). According to the respondents, the help of an energy expert is necessary to supervise the works. The EEM appears to be a useful tool to facilitate the entry of young families into the real estate market for sustainable buildings. Initiatives that promote discounts on taxes that affect buildings (such as a waste tax) can support the choice of efficient properties even if to a lesser extent. Responses to follow-up questions confirm that individuals value the increased monetary savings generated by energy efficiency and the many co-benefits provided, such as increased comfort and health conditions. One reason for the popularity of this type of tool among survey participants is that green lines of credit can often be combined with improvements to increase their appeal to customers. They can be combined with grants to help alleviate the upfront cost barrier and can be offered along with guarantees, such as loan loss reserves, to reduce the risk of customer default for lenders and help more customers to access them. In addition, EEM would allow potential purchasers to target deeper restructuring by having more capital at their disposal. Homeowners can achieve an economic return over time, guaranteed by the energy savings provided by the investments. The results of the evaluation can help banks to understand if this innovative financing model can partly drive mortgage applications, which have gone through a period of slowdown. As for decision-makers, our results can inform the design of policy incentives that are complementary to those provided by EEM, thereby avoiding the introduction of incentive schemes that are overly costly to society as a whole. The results of the study could also be relevant in the context of the recent EU taxonomy for sustainable activities that came into force on 31 December 2021. Bearing in mind the scope of the EU’s climate and energy objectives, the EU taxonomy represents a significant step forward in developing a new market paradigm that places the financial services industry at the heart of efforts to increase sustainable investment and implement the EU Green Deal (Bottero & Dell’Anna, 2022). In this context, the EEM, by pursuing the implementation of a standardized credit scheme for European countries, aims to define a new market ecosystem that combines private sector finance with public sector support, to ensure the best solutions for consumers, lenders, and investors and the economy as a whole. Since EEM was studied in this study from the perspective of private residential investors, it would be interesting to determine in the future whether this financial tool could support companies in sustainable financial goals based on environmental, social, and governance criteria.

## Supplementary Information

Below is the link to the electronic supplementary material.

Supplementary file1 (DOCX 868 KB)

Supplementary file2 (DOCX 30.2 KB)

## Footnotes

1 The ten Italian banks participating in the energy efficient mortgages pilot project, offering energy-efficiency mortgages are Banco Bpm, Bnl-Bnp Paribas, Bper Banca, Cassa Centrale-Credito Cooperativo Italiano, Crédit Agricole-Cariparma, Friulovest Banca, Monte dei Paschi, the Italian branch of Société Générale, UniCredit, and Volksbank Alto Adige.
