# Supplementary material for: A choice experiment for testing the energy-efficiency mortgage as a tool for promoting sustainable finance
Source: Energy Effic. 2022 May 4;15(5):27. doi: 10.1007/s12053-022-10035-y (PMC9064716; doi:10.1007/s12053-022-10035-y)
Supplement: Supplementary file 1 — Supplementary file1 (DOCX 868 KB) [file 12053_2022_10035_MOESM1_ESM.docx]

***Dell’Anna F., Marmolejo-Duarte C., Bravi M., Bottero M. (2022), A Choice Experiment for testing Energy-Efficiency Mortgage as a tool for promoting sustainable finance, Energy Efficiency, Springer, doi:10.1007/s12053-022-10035-y***

**Appendix 1**

**Supporting information 1** Questionnaire based on the choice experiment approach to investigate the preferences of young families in buying a new house.

**Questionnaire**

We are studying the preferences of families when they a buying a new home. If you have 10 minutes to spare, please answer the questionnaire.

The survey is for people over the age of 18.

The questionnaire is anonymous.

Thanks for your participation!

**SECTION 1 – CHOICE EXPERIMENT**

First, we will introduce you to the alternatives that can characterize a dwelling. Next, we will ask you to choose the combination you prefer.


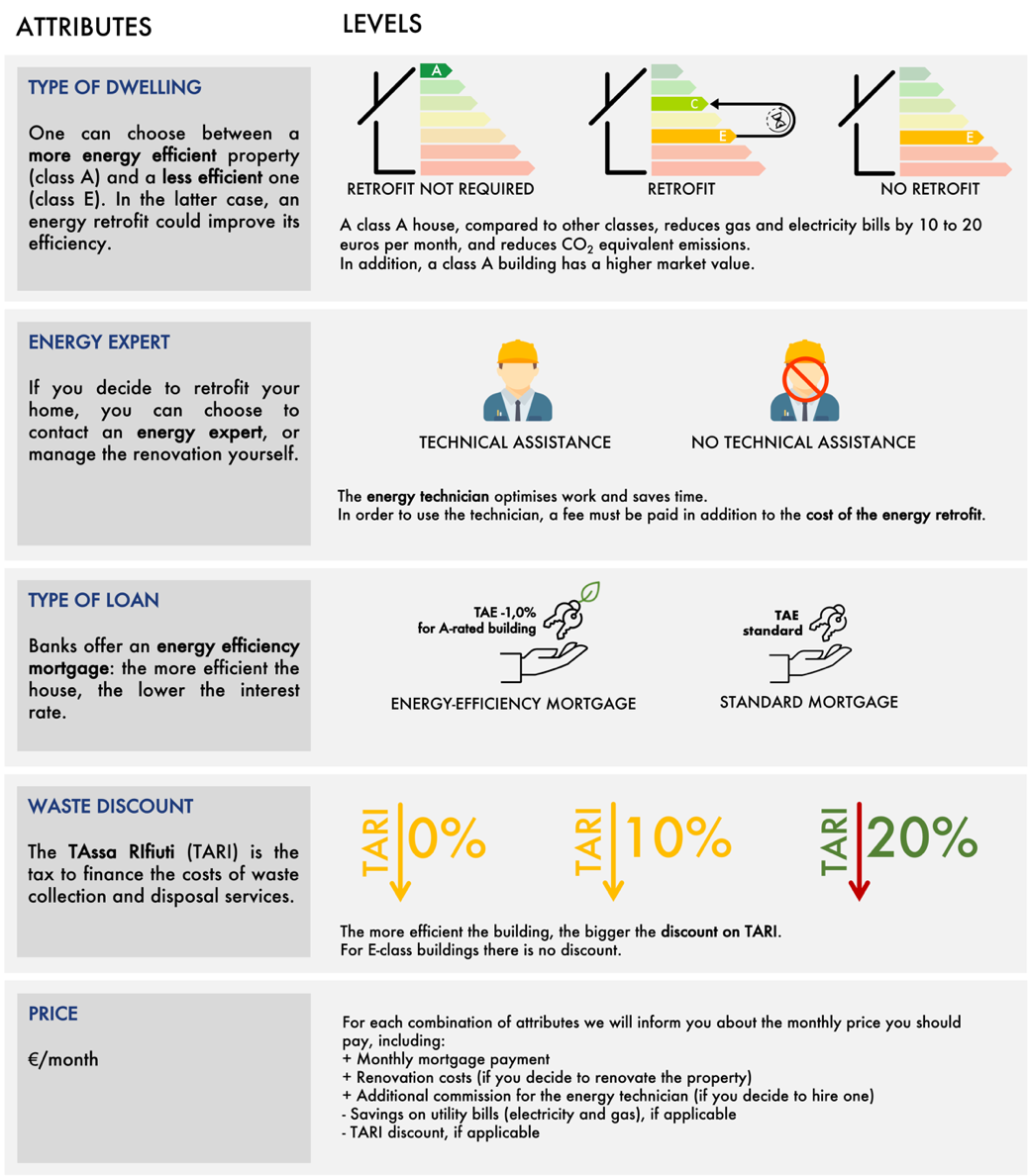


Please choose the home you would buy in each of the following four scenarios.

1.1 If you had to buy a home, which of the following combinations would you choose? *(Example of a random choice task.)*

**
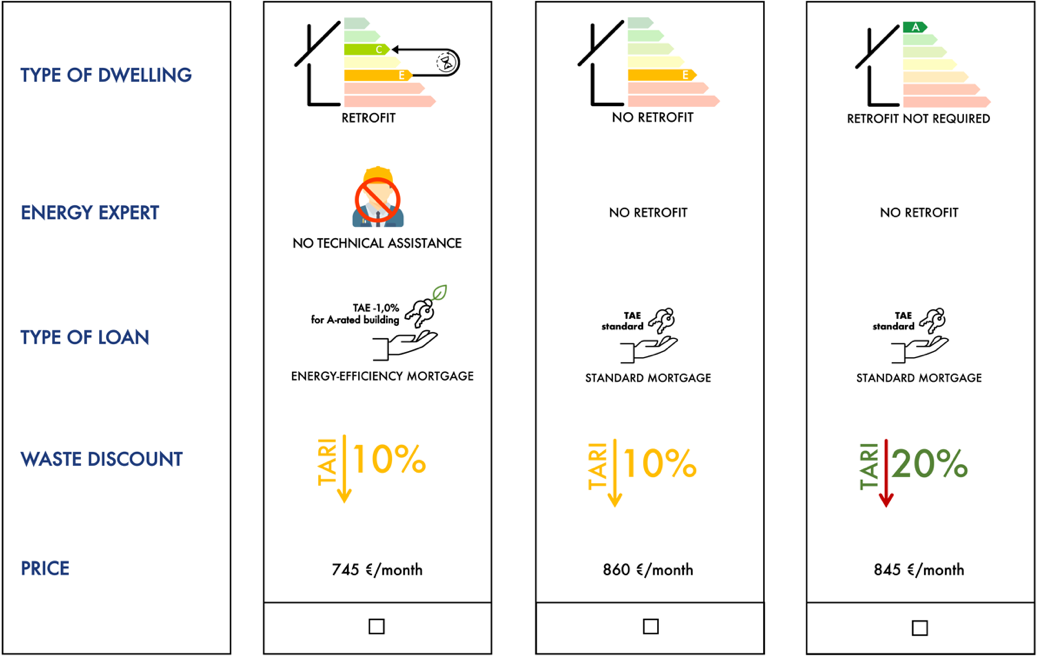
**

1.2 If you had to buy a home, which of the following combinations would you choose? *(Example of a random choice task.)*

**
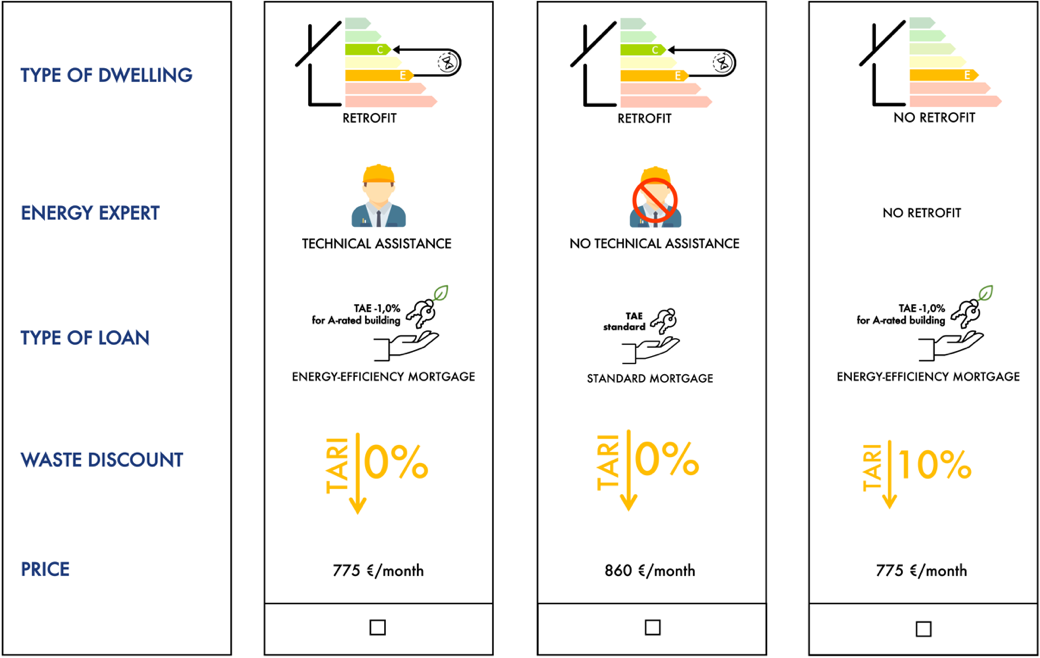
**

- 1. If you had to buy a home, which of the following combinations would you choose? *(Example of a random choice task.)*

**
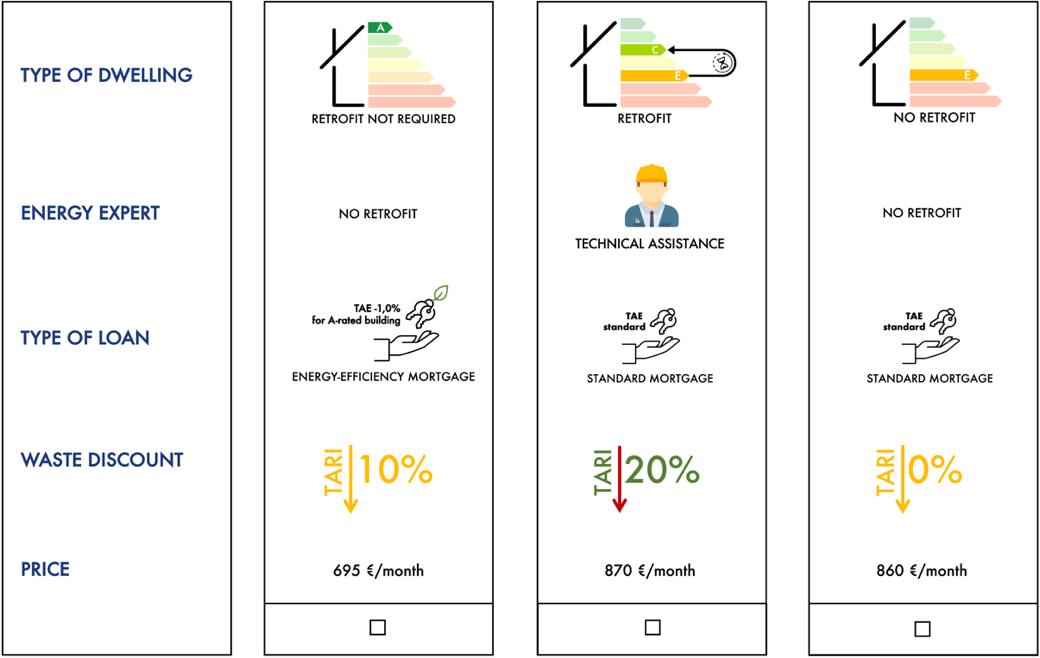
**

- 1. If you had to buy a home, which of the following combinations would you choose? *(Example of a random choice task.)*

**
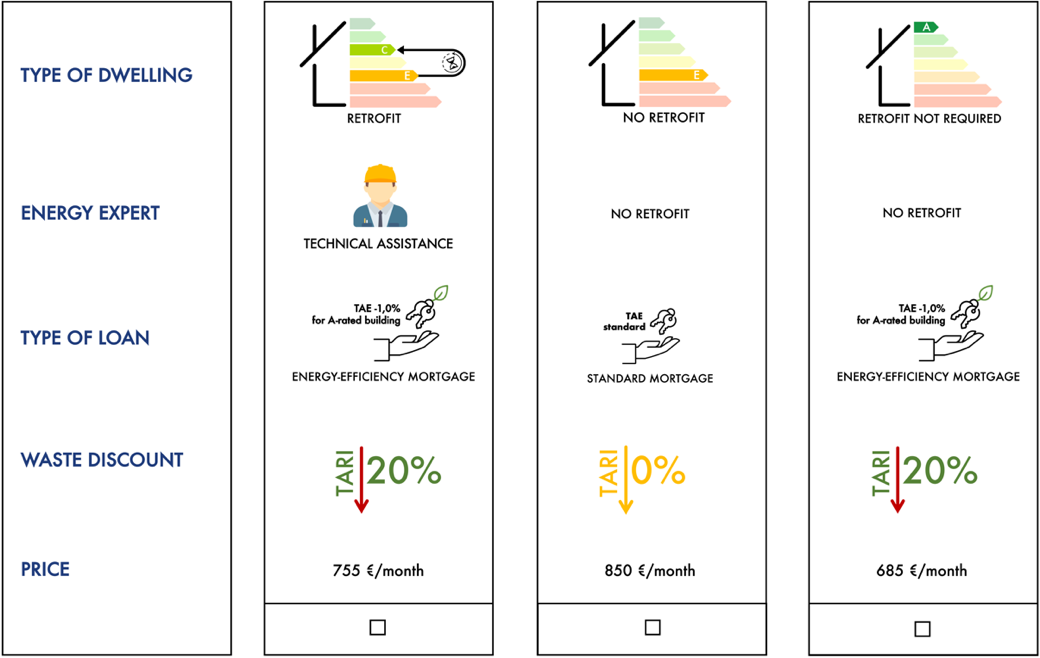
**

**SECTION 2 - ENERGY KNOWLEDGE**

2.1 How important do you consider these possible benefits of energy efficiency to be?

|  | *Not important* | *Less important* | *Important* | *Very important* |
| --- | --- | --- | --- | --- |
| Bill reduction | ☐ | ☐ | ☐ | ☐ |
| Waste tax reduction | ☐ | ☐ | ☐ | ☐ |
| Loan interest rate reduction | ☐ | ☐ | ☐ | ☐ |
| Market value increase | ☐ | ☐ | ☐ | ☐ |
| Indoor comfort increase | ☐ | ☐ | ☐ | ☐ |
| Environmental impact reduction | ☐ | ☐ | ☐ | ☐ |
| Health condition increase | ☐ | ☐ | ☐ | ☐ |

- 1. Renovating a dwelling has advantages and disadvantages. How do you evaluate each of them?

|  | *Not important* | *Less important* | *Important* | *Very important* |
| --- | --- | --- | --- | --- |
| Personalizing the home | ☐ | ☐ | ☐ | ☐ |
| Restructuring inconvenience | ☐ | ☐ | ☐ | ☐ |
| Energy expert employment | ☐ | ☐ | ☐ | ☐ |

- 1. How reliable are the following institutions in encouraging the purchase of efficient housing?

|  | *Not reliable* | *Less reliable* | *Reliable* | *Very reliable* |
| --- | --- | --- | --- | --- |
| Public institutions | ☐ | ☐ | ☐ | ☐ |
| Banks | ☐ | ☐ | ☐ | ☐ |

**SECTION 3 - INTERVIEWED ATTITUDES**

3.1 The house where you currently live is...

- Owned
- Owned (I’m paying a mortgage)
- Rented
- Not owned/rented
  1. Let us assume that you are the owner of the house where you live, and you wanted to invest in some improvements, what would be your priority?

|  | *Very low* | *Low* | *High* | *Very high* |
| --- | --- | --- | --- | --- |
| Bathroom and kitchen makeover | ☐ | ☐ | ☐ | ☐ |
| Internal distribution | ☐ | ☐ | ☐ | ☐ |
| Envelope insulation | ☐ | ☐ | ☐ | ☐ |
| Windows replacement | ☐ | ☐ | ☐ | ☐ |
| Boiler replacement | ☐ | ☐ | ☐ | ☐ |

- 1. If you had to move house, would you prefer...
- To buy a property
- To rent a property
  1. Please indicate how often you perform these actions:

|  | *Very low* | *Low* | *High* | *Very high* |
| --- | --- | --- | --- | --- |
| Recycling | ☐ | ☐ | ☐ | ☐ |
| Purchasing efficient appliances | ☐ | ☐ | ☐ | ☐ |
| Energy savings at home | ☐ | ☐ | ☐ | ☐ |

- 1. Please indicate how much you believe that the following aspects influence the energy bill.

|  | *Not much* | *Much* | *I don’t know* |
| --- | --- | --- | --- |
| Envelope insulation | ☐ | ☐ | ☐ |
| Heating and cooling | ☐ | ☐ | ☐ |
| Windows typology | ☐ | ☐ | ☐ |
| Shading system | ☐ | ☐ | ☐ |
| Occupant behaviour | ☐ | ☐ | ☐ |

- 1. Select the option that seems most appropriate to you.

|  | *True* | *False* | *I don’t know* |
| --- | --- | --- | --- |
| Investing in government bonds is riskier than investing in the stock market | ☐ | ☐ | ☐ |
| In the long term, the interest rates on fixed mortgages are higher than the variable rates | ☐ | ☐ | ☐ |
| A more profitable investment involves greater risks | ☐ | ☐ | ☐ |

**SECTION 4 – PERSONAL INFORMATION**

4.1 What is your level of educational attainment?

- Primary school
- Secondary school
- Upper secondary school
- Bachelor’s degree
- Master’s degree
- Postgraduate degree
  1. Please indicate your age group:
- Less than 18 years old
- 18–24 years
- 25–34 years
- 35–44 years old
- 45–65 years old
- Over 65 years old
  1. Indicate the net income level of your household (remember that the questionnaire is anonymous)
- less than 600 €/month
- 601–1200 €/month
- 1201–1800 €/month
- 1801–2400 €/month
- 2401–3600 €/month
- 3601–4800 €/month
- more than 4800 €/month
  1. Job:
- Student
- Worker
- Retiree
- Housewife
- Unemployed
  1. Gender
- Male
- Female
  1. Please indicate the postcode of your residence (‌five digits)

_ _ _ _ _

Thanks for participating in the survey.
