# Supplementary material for: A choice experiment for testing the energy-efficiency mortgage as a tool for promoting sustainable finance
Source: Energy Effic. 2022 May 4;15(5):27. doi: 10.1007/s12053-022-10035-y (PMC9064716; doi:10.1007/s12053-022-10035-y)
Supplement: Supplementary file 2 — Supplementary file2 (DOCX 30.2 KB) [file 12053_2022_10035_MOESM2_ESM.docx]

***Dell’Anna F., Marmolejo-Duarte C., Bravi M., Bottero M. (2022), A Choice Experiment for testing Energy-Efficiency Mortgage as a tool for promoting sustainable finance, Energy Efficiency, Springer, doi:10.1007/s12053-022-10035-y***

**Appendix 2**

**Supporting information Table B1** Descriptive analysis of the socio-economic characteristics of the individuals in the sample.

**Supporting information Table B2** Descriptive analysis of knowledge of the individuals in the sample about the effects of energy efficiency.

**Supporting information Table B3** Descriptive analysis of survey respondents' homes.

**Supporting information Table B4** Descriptive analysis of respondents' sustainability attitudes.

**Supporting information Table B5** Descriptive analysis of individuals’ energy and financial knowledge

Table B1. Sample’s descriptive statistics

| Variable | Level | Absolute frequency | Relative frequency (%) |
| --- | --- | --- | --- |
| Gender (GEN) | Male | 93 | 54.6 |
|  | Female | 112 | 45.4 |
| Age (AGE) | 18–24 | 65 | 31.7 |
|  | 25–34 | 85 | 41.5 |
|  | 35–44 | 55 | 26.8 |
|  |  |  |  |
| Educational level (EDU) | Primary school | 0 | 0 |
|  | Secondary school | 5 | 2.4 |
|  | Upper secondary school | 97 | 47.3 |
|  | Bachelor’s degree | 42 | 20.5 |
|  | Master’s degree | 38 | 18.5 |
|  | Postgraduate degree | 23 | 11.2 |
| Occupation | Student | 75 | 36.6 |
|  | Worker | 116 | 56.6 |
|  | Housewife | 6 | 1.5 |
|  | Unemployed | 11 | 5.4 |
| Family monthly income (INC) | < 600 € | 5 | 2.4 |
|  | 601–1200 € | 28 | 13.7 |
|  | 1201–1800 | 62 | 30.2 |
|  | 1801–2400 | 32 | 15.6 |
|  | 2401–3600 | 49 | 23.9 |
|  | 3601–4800 | 14 | 6.8 |
|  | > 4800 | 15 | 7.3 |

Table B2. Energy efficiency actions and individuals’ knowledge from the sample

| *Energy efficiency impact* | *Not important* | *Less important* | *Important* | *Very important* |
| --- | --- | --- | --- | --- |
| Bill reduction | 0 | 7 | 85 | 113 |
|  | (0) | (3.4) | (41.5) | (55.1) |
| Waste tax reduction | 4 | 30 | 93 | 78 |
|  | (2) | (14.6) | (45.4) | (38) |
| Loan interest rate reduction | 3 | 24 | 90 | 89 |
|  | (1) | (11.7) | (43.9) | (43.4) |
| Market value increase | 4 | 31 | 88 | 82 |
|  | (2) | (15.1) | (42.9) | (40) |
| Indoor comfort increase | 3 | 14 | 92 | 96 |
|  | (1.5) | (6.8) | (44.9) | (46.8) |
| Environmental impact reduction | 3 | 14 | 66 | 122 |
|  | (1.5) | (6.8) | (32.2) | (59.5) |
| Health condition increase | 0 | 6 | 65 | 134 |
|  | (0) | (2.9) | (31.7) | (65.4) |
| *Retrofit operations impact* | *Not important* | *Less important* | *Important* | *Very important* |
| Restructuring customization | 0 | 14 | 105 | 86 |
|  | (0) | (6.8) | (51.2) | (42) |
| Disruption of the restructuring | 15 | 94 | 72 | 24 |
|  | (7.3) | (45.9) | (35.1) | (11.7) |
| Energy expert employment | 9 | 43 | 94 | 59 |
|  | (4.4) | (21) | (45.9) | (28.8) |
| *Reliability of institutions* | *Not reliable* | *Less reliable* | *Reliable* | *Very reliable* |
| Public institutions | 16 | 100 | 74 | 15 |
|  | (7.8) | (48.8) | (36.1) | (7.3) |
| Banks | 18 | 87 | 88 | 12 |
|  | (8.8) | (42.4) | (42.9) | (5.9) |

Table B3. Individuals’ property description

|  | *Owned* | *Owned (I’m paying a mortgage)* | *Rented* | *Not owned/rented* |
| --- | --- | --- | --- | --- |
| House ownership | 64 | 30 | 60 | 51 |
|  | (31.2) | (14.6) | (29.3) | (24.9) |
| *Retrofit priority* | *Very low* | *Low* | *High* | *Very high* |
| Bathroom and kitchen makeover | 30 | 67 | 76 | 32 |
|  | (14.6) | (32.7) | (37.1) | (15.6) |
| Internal distribution | 34 | 89 | 61 | 21 |
|  | (16.6) | (43.4) | (29.8) | (10.2) |
| Envelope insulation | 19 | 55 | 69 | 62 |
|  | (9.3) | (26.8) | (33.7) | (30.2) |
| Windows replacement | 25 | 46 | 69 | 65 |
|  | (12.2) | (22.4) | (33.7) | (31.7) |
| Boiler replacement | 21 | 39 | 70 | 75 |
|  | (10.2) | (19) | (34.1) | (36.6) |
| *Individual preference* | *buy a property* | | *rent a property* | |
| You would prefer to... | 176 | | 39 | |
|  | (85.9) | | (14.1) | |

Table B4. Individuals’ sustainable attitudes

| *Sustainable actions* | *Very low* | *Low* | *High* | *Very high* |
| --- | --- | --- | --- | --- |
| Recycling | 4 | 11 | 55 | 135 |
|  | (2) | (5.4) | (26.8) | (65.9) |
| Purchase efficient appliances | 12 | 56 | 86 | 51 |
|  | (5.9) | (27.3) | (42) | (24.9) |
| Energy savings at home | 6 | 18 | 95 | 86 |
|  | (2.9) | (8.8) | (46.3) | (42) |

Table B5. Individuals’ energy and financial knowledge

| *The energy bill is influenced by* | *Not much* | *Much* | *I don’t know* |
| --- | --- | --- | --- |
| Envelope insulation | 21 | 162 | 22 |
|  | (10.2) | (79) | (10.7) |
| Heating and cooling | 11 | 182 | 12 |
|  | (5.4) | (88.8) | (5.9) |
| Windows typology | 22 | 165 | 18 |
|  | (10.7) | (80.5) | (8.8) |
| Shading system | 103 | 76 | 26 |
|  | (50.2) | (37.1) | (12.7) |
| Occupant behaviour | 17 | 173 | 15 |
|  | (8.3) | (84.4) | (7.3) |
| *Financial knowledge* | *True* | *False* | *I don’t*  *know* |
| Investing in government bonds is riskier than investing in the stock market | 15 | 88 | 102 |
|  | (7.3) | (42.9) | (49.8) |
| In the long term, the interest rates on fixed mortgages are higher than the variable rates | 61 | 46 | 98 |
|  | (29.8) | (22.4) | (47.8) |
| A more profitable investment involves greater risks | 115 | 40 | 50 |
|  | (56.1) | (19.5) | (24.4) |
